# Class imbalance learning with Bayesian optimization applied in drug discovery

**DOI:** 10.1038/s41598-022-05717-7

**Published:** 2022-02-08

**Authors:** Shenmin Guan, Ning Fu

**Affiliations:** Shanghai GenomSeqCare Biotechnology Co. Ltd., Shanghai, 200052 China

**Keywords:** Drug discovery, Drug screening

## Abstract

Machine intelligence (MI), including machine learning and deep learning, have been regarded as promising methods to reduce the prohibitively high cost of drug development. However, a dilemma within MI has limited its wide application: machine learning models are easier to interpret but yield worse predictive performance than deep learning models. Therefore, we propose a pipeline called Class Imbalance Learning with Bayesian Optimization (CILBO) to improve the performance of machine learning models in drug discovery. To demonstrate the efficacy of the CILBO pipeline, we developed an example model to predict antibacterial candidates. Comparison of the antibacterial prediction performance between our model and a well-known deep learning model published by Stokes et al. suggests that our model can perform as well as the deep learning model in drug activity prediction. The CILBO pipeline we propose provides a simple, alternative approach to accelerate preliminary screenings and decrease the cost of drug discovery.

## Introduction

Drug development is a costly process that takes more than 10 years and up to an average of 2.6 billion USD to bring a drug from preliminary discovery to the market^1–5^. Much of this cost is attributed to the high attrition rate of the failure trials of the candidate drugs^3,4^. Despite substantial investment in the selection of candidate molecules from hundreds or thousands of compounds, the success rate for new candidates to finally reach the market is approximately 10%^4^. Therefore, selecting the most promising candidate molecules will help to accelerate the research process and reduce final stage failure, thereby minimizing the cost of drug development.

Machine intelligence (MI), including machine learning and deep learning, has been successfully applied to drug discovery and is regarded as a promising method for such candidate selection^6,7^. However, there is a dilemma between performance and interpretability within MI^8^, which has limited its application. Previous studies have shown that deep learning models perform better than machine learning models on classifications^9,10^ but are harder to interpret^11^. The reason deep learning models are more difficult to interpret is that it is harder to find direct and reliable correlations between features the models utilized in classification and the output predictions^11^. These limitations are unfavourable in drug development because researchers in the field would prefer to acquire not only the capacity for prediction but also the knowledge suggested by the model^12^.

Developing methods to interpret the outcomes of deep learning models is no simple task^12–14^. Instead, improving the performance of machine learning models may offer a faster and easier solution to alleviate the dilemma of predictivity and interpretability. For instance, automatic machine learning (AutoML) is one promising strategy to enhance the drug development process.

Models built on automatic machine learning have been verified in healthcare to have relatively good performance^15,16^. Even the deep learning model's performance sometimes relies on the network structure suggested by automatic machine learning^10^. Under the AutoML algorithm, various hyperparameters or ensemble methods can be automatically tried tens to hundreds of times to improve the performance of the machine learning model. Meanwhile, the AutoML algorithm frees the researcher from these tedious, and repetitive trial and error as well^17^. Besides, training a machine learning model generally takes orders of magnitude less time than training a deep learning model. Machine learning models built on AutoML can be easily trained with many more hyperparameters in an acceptable training time.

Additionally, drug discovery datasets are usually highly imbalanced, that contain very few functional candidates (interested class) and hundreds or thousands of times more unfunctional molecules (uninterested class)^18,19^. The minority interested class is more likely to be predicted as a rare occurrence, ignored altogether, or assumed as noise or an outlier, which causes bias and leads to poor generalization performance^20^. Although several previous studies in disease and drug related fields proved that addressing class imbalance problem appropriately would improve the model’s performance^21–23^, this problem has still been frequently ignored. Once the machine learning models apply strategies to minimize classification bias caused by the imbalanced datasets, it may further benefit the performance improved by the AutoML algorithm.

Therefore, we suggest a pipeline of constructing a machine learning model using Bayesian Optimization with strategies for imbalanced datasets to improve the classification performance of this model on drug discovery. In this pipeline, the best hyperparameter combination of the model variables, training and treating imbalanced datasets is suggested with Bayesian optimization. Bayesian optimization is a sequential design strategy for global optimization of black-box functions that does not assume any functional forms, and it seems particularly well-positioned for the application areas like drug development^24,25^. The optimization used here differs from commonly used hyperparameter optimization by addressing the issue of class imbalance. We call this pipeline Class Imbalance Learning with Bayesian optimization (CILBO). Antibiotic predictions are used here as an example to evaluate the effectiveness of this pipeline.

In the past decades, antibiotic screening has become prohibitively costly and has decreased dramatically^10,26–29^. This situation, together with widespread antibiotic resistance, makes the discovery of new antibiotics critical^10,26,30^. A recent work done by Stokes et al. showed the successful discovery of new antibiotics through the combination of a graph neural network (GNN) model prediction and empirical investigation^10^. Stokes’ research highlighted the significant impact of deep learning on early antibiotic discovery, but interpreting the results generated by their model is still not an easy task.

Here, using the same datasets described in the paper by Stokes et al.^10^, we generated a random forest classifier with CILBO and compared the predictions of our model to those of Stokes’ model^10^. The comparison suggests that our machine learning model can perform predictions as well as the deep learning model. The CILBO pipeline is simple to run and able to efficiently improve the performance of machine learning models, offering an alternative approach that can be widely applied in many fields of drug discovery.

## Results

### Summary of our example model construction

**Figure 1:**
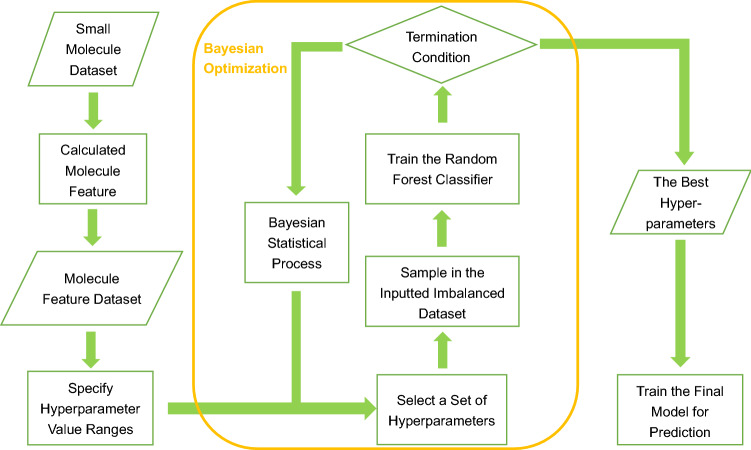
Workflow of the final model construction.

To improve the classification performance of the easy-to-interpret machine learning models on drug discovery, we built a pipeline called Class Imbalance Learning with Bayesian Optimization (CILBO). This pipeline uses Bayesian optimization to suggest the best combination of hyperparameters for model variables, training, and treating imbalanced datasets of a machine learning model. We constructed a random forest classifier based on CILBO as an example to verify CILBO’s effectiveness on improving model performance in drug discovery (Fig. 1).

### Evaluation of our model performance during the training phase

We trained our model on the same datasets used in Stokes’ GNN model^10^. The dataset used for training contained 2335 molecules, but only 120 of them had proved antibacterial activity (see Supplementary Table S1). The dataset was highly imbalanced. To compare the performance and outcomes of our model with Stokes’ deep learning model, we used the same training dataset and training/testing ratio (see “Methods” section).

The best hyperparameters suggested by Bayesian optimization, which were used in this study, are listed in Table 1. The last two hyperparameters, “class_weight” and “sampling_strategy” were utilized to minimize the bias caused by dataset imbalance and enhance the overall model performance. The feature “fingerprint” providing the descriptions of the topological structure representations was very useful for interpreting the model. The RDK fingerprint computed by RDKit^31^ was selected as the molecule feature for our model because it behaved optimally compared to other optional features (for descriptors and other fingerprints, see “Methods” section).

**Table 1:**
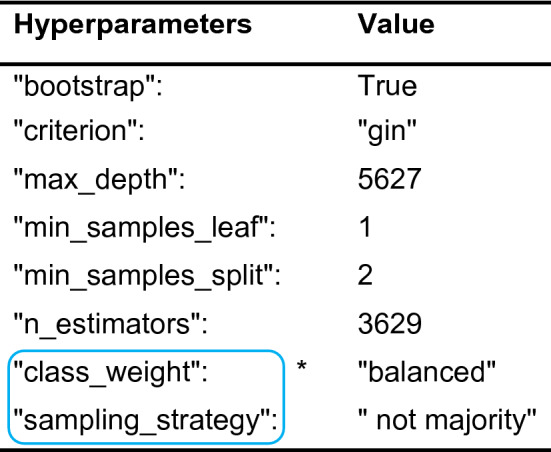
Best hyperparameters suggested by Bayesian optimization.

*Frame indicates the hyperparameters for treating imbalanced datasets.

**Figure 2:**
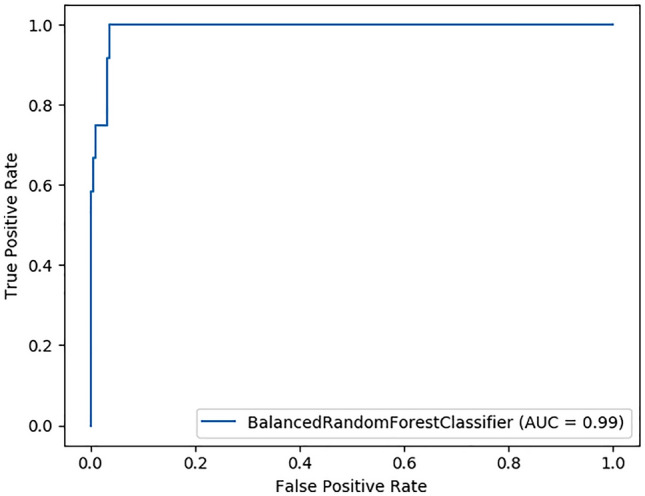
ROC-AUC of our final model.

With these best hyperparameters and feature, the average receiver operating characteristic curve-area under the curve (ROC-AUC) of our model, after 30 times fivefold cross-validation at the training phase, was about 0.917 (Supplementary File random_forest_antibiotic_20210511c.py). It was greater than 0.896, by the ROC-AUC of Stokes’ model^10^. After being enhanced by the best hyperparameters and molecule features described above, and with more training samples (the training set includes 90% of molecules), our final model achieved a ROC-AUC of 0.99 (Fig. 2). The confusion matrix based on the test set of our final model is shown in Table 2. According to this matrix, our model did not classify any non-antibacterial molecules as antibacterial, which suggests that the model has a low false-positive rate for identifying candidate compounds.

**Table 2:** Confusion matrix of our final model.

Predicted	Actual	
	Non-antibacterial	Antibacterial
Non-antibacterial	221	0
Antibacterial	5	7

This confusion matrix is based on testing set of our final model, molecules with prediction score above 0.5 were regarded as predicted antibacterials.

### Comparison of the prediction results generated by our model and Stokes’ model in antibacterial discovery

Our final model was then applied to identify candidate molecules with antibacterial characteristics from the library recorded by the Drug Repurposing Hub^10,32^. This library contained 6111 molecules at different stages of investigation for human diseases. After removing compounds with molecular graphs common between the training dataset (2335 molecules) and the Drug Repurposing Hub (6111 molecules), a dataset containing 4496 unique molecules was used for the identification. Importantly, this dataset was also the same set of molecules used by Stokes et al. to demonstrate their model^10^. For each molecule, our model provides a prediction score. The full list of prediction results can be found in Supplementary Table S2.

**Figure 3:**
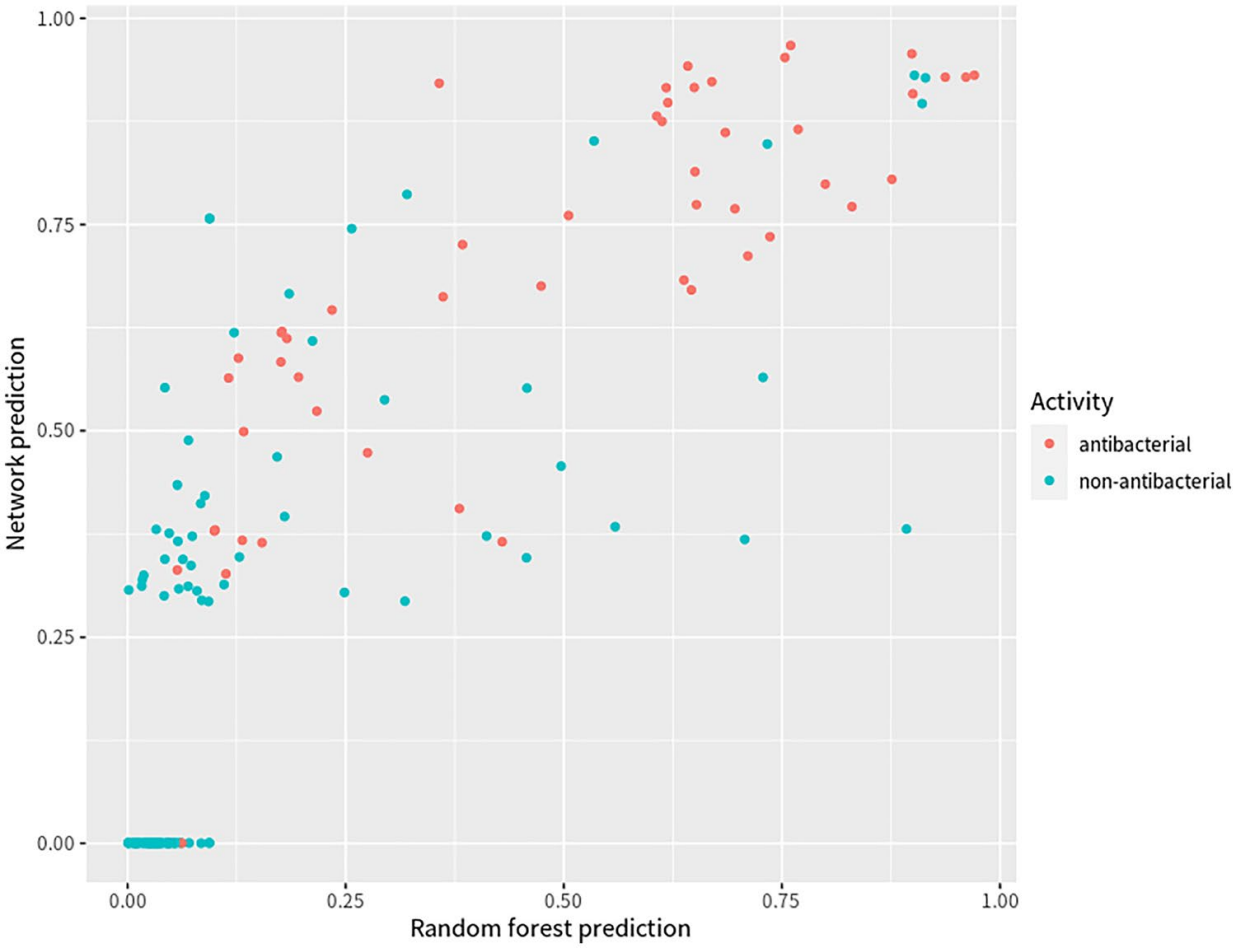
Plot of the prediction results by both models. Plot of the 162 molecules with empirically tested antibacterial information and also predicted with top and bottom scores respectively for antibacterial properties by Stokes’ model^10^. Blue dots represent non-antibacterials; orange dots represent antibacterials. X-axis (Pred_Score_Forest) is the score predicted by our final model, a random forest classifier; Y-axis (Pred_Score_Net) is the score predicted by Stokes’ final model^10^, a graph neural network.

Using their model, Stokes and his colleagues selected the top 99 molecules most strongly predicted to display antibacterial properties, along with the 63 molecules with the lowest prediction scores. To validate this finding, Stokes et al. empirically tested these 162 (99 + 63) molecules^10^. We leveraged Stokes’ empirically tested antibacterial information to estimate our own prediction results across the same set of 162 molecules (Supplementary Table S3). We found that our model was comparably effective as the Stokes’ model in its prediction of the 162 molecules with antibacterial properties. (Fig. 3).

Specifically, about 75% of the molecules with prediction scores above 0.5 in our model (model default threshold score) were found to be empirically tested antibacterials, while 74% of the molecules with prediction scores above 0.5 in the Stokes’ model were empirically tested antibacterials. When a threshold score above 0.5 was selected in both models, about 80% of molecules meeting the criteria had empirically tested antibacterial properties, which was higher than the number by any model alone. If requires 90% of the empirically tested antibacterial molecules were screened out, the score threshold of our model and Stokes’s model would be 0.15 and 0.3 respectively based on this comparison (Fig. 3). Of the molecules that scored near zero, almost all were absent of antibacterial properties in both models. Again, this finding further emphasizes that our model is comparable with the Stokes’ model in its prediction capacity.

## Discussion

Machine Intelligence (MI) has been regarded as a promising approach to help relieve the pressure of prohibitively costly procedures during drug discovery. However, the dilemma between predictivity and interpretability within MI has limited broader applications in drug discovery. Therefore, we suggested a pipeline Class Imbalance learning with Bayesian Optimization (CILBO) to improve the machine learning model’s classification performance.

We verified the performance of an example model built based on CILBO for candidate antibiotic discovery. Here, a random forest classifier constructed using Bayesian optimization with strategies for imbalanced datasets was applied as the example model. The random forest classifier was adopted because it limits overfitting and is easy to interpret.

By using the same datasets and similar training conditions described in Stokes’ paper^10^ (see “Methods” section), our model was comparable to Stokes’ model. Based on the same splitting ratio of training and testing sets, the average ROC-AUC of our model could reach 0.917, which is notably higher than 0.896 the ROC-AUC of Stokes’ model. Meanwhile, the results of imbalanced random forest model with empirical hyperparameters and xgboost were also compared with our model, and the average ROC-AUC calculated based on them were 0.895 and 0.901 respectively (see the Supplementary File “machine_learning_model_20211208.py”). This finding indicates that the performance of our model is not inferior to that of Stokes’ deep learning model^10^ based on the comparatively simple library we used for training.

When directly comparing the prediction results of two models among the 162 empirically tested molecules, our model also successfully captured promising candidates at a rate comparable to Stokes’ deep learning model. These results all suggest that our model can perform as well as deep learning models in early screenings for drug discovery.

A key natural benefit of the CILBO model is that it is easy to interpret. Furthermore, the time required to train a machine learning model like the random forest model used here is at least 100 times shorter than the time required to train a deep learning model. It provides enough time to try various hyperparameters automatically and identify the best ones to enhance the model. Another obvious advantage of the model built on CILBO compared to the normal deep learning model is that CILBO is less dependent on the model designer and infrastructure. To design a neural network model, the researcher needs a rich experience and a high-end machine. The random forest model is simpler to design since the key characters are provided by Bayesian optimization, an AutoML method. Therefore, the simplicity and efficiency of our model will make it applicable in a wider context of drug candidate selection.

Of particular note is that imbalanced datasets are fairly common in the drug industry. They may cause severe classification bias during MI aided drug development, but this problem has been frequently ignored. We considered this imbalance problem in the construction of our model and used special hyperparameters to control for this type of bias. It is designed to enhance the performance of our model.

In our work, we constructed a special random forest model using CILBO (a pipeline we proposed), and compared this machine learning model to the deep learning model created by Stokes et al.^10^ in antibiotic discovery. The comparison results together with other properties of our model suggest that: (1) the machine learning model built on CILBO can perform prediction at least as well as the deep learning model; (2) it is naturally easier to interpret and comparatively simpler to operate without requiring a high level of researcher experience, (3) inclusion of strategies for class imbalance to control classification bias further improved the predictive performance of the model built on CILBO, and may broaden its applicability in drug development. Therefore, CILBO, the pipeline we designed provides an alternative and simple solution to promote MI in drug development.

## Methods

### Model selection

We used a random forest model as the classifier in this work (Fig. 1). A random forest model is robust against overfitting^33^ and easy to interpret because routine methods can be used to estimate the importance of and interaction between features^34^.

### Molecule feature selection

The optional molecule features include descriptors, RDK fingerprint, MACCS key, Avalon fingerprint, ECFP4 and ECFP6, which were calculated by RDKit 2020.09.1.0^31^. All of these features were tested separately during the training phase of our model, but not the complex combination of features. Descriptors usually provide information on different molecular properties, such as general physical properties, electrochemical properties and electron cloud characteristics. Fingerprint provides descriptions of various topological structure representations of molecules. RDK fingerprint was chosen for the final model. RDK fingerprint calculated for the dataset used to train our model (including 2335 unique compounds) and the dataset used to predict candidates (including 4496 unique compounds) can see in Tables S4 and S5 respectively.

### Hyperparameters optimization

Bayesian optimization was used to find the best hyperparameters to our model. In this work, Bayesian optimization was used not just for the classifier, but also for the strategies specially dealing with imbalanced datasets. As we observed, the training dataset was highly imbalanced, which may introduce classification bias. Bayesian optimization was supposed to give the best combination of hyperparameters for the classifier and to mitigate the problem caused by class imbalance. This is distinct from common models using an automatic machine learning algorithm. In most cases, only classifier hyperparameters and training details of the models are focused, while strategies to deal with imbalanced datasets are frequently ignored.

Specifically, Python packages, scikit-learn 0.23.2, imbalanced-learn 0.7.0 and scikit-optimize 0.8.1 were used to search for the best hyperparameters. For convenience, the BalancedRandomForestClassifier defined in imbalanced-learn 0.7.0 package was adopted, since it included the sampling strategy and class weight. In our model, the maximum feature number was the default value in BalancedRandomForestClassifier. The hyperparameters that needed to be optimized were as follows:

**Table:** 

Hyperparameters	Value type (range)
n_estimators	Integer (5, 5000)
Criterion	Categorical ([“gini”, “entropy”])
max_depth	Integer (1, 6000)
min_samples_split	Integer (2, 200)
min_samples_leaf	Integer (1, 200)
Bootstrap	Categorical ([True, False])
class_weight	Categorical ([“balanced”, “balanced_subsample”, None])
sampling_strategy	Categorical ([‘majority’, ‘not minority’, ‘not majority’])

The hyperparameters most frequently used in random forest models included: “n_estimators”, “criterion”, “max_depth”, “min_samples_split”, “min_samples_leaf” and “bootstrap”. The last two hyperparameters, “class_weight” and “sampling_strategy”, were specifically used to handle imbalanced datasets.

In Bayesian searching, the ROC-AUC was the metric used for the random forest classifier. BayesSearchCV from the package scikit-optimize was the function for Bayesian searching, and the surrogate model was used by default. The cross-validation folds were created by StratifiedFold in scikit-learn package. The splitting number was set to 5 and the iteration number was 150. These numbers were set according to the relative description in Stokes’ paper^10^ for a better comparison. Once the best hyperparameters were identified, an additional cross validation was performed. The final model was then trained with the best hyperparameters suggested by Bayesian optimization.

### Datasets for training, analysis and direct comparison

The dataset used to train our model was the same dataset described in Stokes’ paper^10^. This dataset combined the molecules from the USFDA-approved Drug Library and those isolated from natural products. It contained 2335 unique compounds, 120 of which had growth inhibitory activity against *E. coli* (see Supplementary Table S1). The dataset was highly imbalanced*.* For a fair comparison, the splitting ratio of training and testing sets used in our model were the same as the Stokes model^10^. It was about 80%/20% (training/testing) for the cross validation, and approximately 90%/10% for the final model training.

The dataset we used for candidate prediction was also the same dataset described by Stokes’^10^. It was a dataset from Drug Repurposing Hub^10,32^ containing 6111 molecules at various stages of investigation for human diseases. By removing the compounds with molecule graphs common between the training set and the Drug Repurposing Hub, the remaining 4496 molecules were used for both our model and Stokes’ model prediction (see Supplementary Table S2). Of these molecules, 99 were predicted the most effectively and 63 were predicted the most ineffectively by the Stokes deep learning model (see Supplementary Table S3). These 162 (99 + 63) molecules were empirically tested for growth inhibition against *E. coli* by Stokes and his colleagues, and 53 (51 out of 99 and 2 out of 63) of them were found to have antibacterial properties.

The dataset used for direct comparison of prediction results between our model and Stokes’ model was based on the 162 molecules with empirically tested antibacterial information.

## Supplementary Information

Supplementary Information 1.

Supplementary Information 2.

Supplementary Tables.
